# Limited Palatal Muscle Resection for the Treatment of Obstructive Sleep Apnea: A Systematic Review and Meta-Analysis

**DOI:** 10.3390/medicina59081432

**Published:** 2023-08-08

**Authors:** Marn Joon Park, Young-Ha Lee, Jae Hoon Cho, Ji Ho Choi

**Affiliations:** 1 Department of Otorhinolaryngology-Head and Neck Surgery, Inha University Hospital, Inha University School of Medicine, 27, Inhang-ro, Jung-gu, Incheon 22332, Republic of Korea; 2 Department of Otorhinolaryngology, Wonju Severance Christian Hospital, Yonsei University Wonju College of Medicine, 20, Ilsan-ro, Wonju 26426, Republic of Korea; 3 Department of Otorhinolaryngology-Head and Neck Surgery, Konkuk University School of Medicine, 120-1, Neungdong-ro, Gwangjin-gu, Seoul 05030, Republic of Korea; 4 Department of Otorhinolaryngology-Head and Neck Surgery, Soonchunhyang University College of Medicine, Bucheon Hospital, 170, Jomaru-ro, Bucheon 14584, Republic of Korea

**Keywords:** obstructive sleep apnea, palatal surgery, PMR, limited palatal muscle resection, meta-analysis, systemic review

## Abstract

*Background and Objectives:* Limited palatal muscle resection (PMR) is a surgical technique employed to alleviate respiratory disturbances in obstructive sleep apnea (OSA) patients with retropalatal narrowing by reducing soft palate volume and tightening the muscles. Although some previous publications have demonstrated the effectiveness of limited PMR, the overall efficacy and therapeutic role of limited PMR for the treatment of OSA remain uncertain. This study utilized meta-analysis and a systematic literature review to estimate the overall effectiveness of limited PMR in treating OSA. *Materials and Methods:* Multiple databases, including PubMed, EMBASE, Cochrane Library, and Web of Science, were searched using specific keywords related to OSA and limited PMR. Original articles assessing respiratory disturbances before and after limited PMR in patients with OSA were included. Data from selected articles were collected using standardized forms, including clinicodemographic characteristics, apnea-hypopnea index (AHI), and lowest pulse oximetry values (minimum SpO_2_). Random effect models were used for analyzing significant heterogeneity. Egger’s test and funnel plot were used to identify publication bias. *Results:* Four studies were included in this meta-analysis for AHI, and three studies were included for minimum SpO_2_ during sleep. A significant reduction in the AHI and an increase in the minimum SpO_2_ were shown following limited PMR as the standardized mean difference (95% confidence interval) was 2.591 (1.092–4.090) and 1.217 (0.248–2.186), respectively. No publication bias was found in either analysis. *Conclusions:* The results of the meta-analysis and systemic review add to the literature that limited PMR can result in a reduction in the AHI and an increase in min SaO_2_. In OSA patients with suspected retropalatal obstruction, limited PMR may be efficiently performed.

## 1. Introduction

Obstructive sleep apnea (OSA) is a common disorder characterized by recurrent upper airway collapse during sleep, leading to intermittent hypoxia, hypercapnia, altered intrathoracic pressure, heightened sympathetic activity, and disrupted sleep [1,2]. OSA prevalence is estimated at 4% to 5% in men and 2% to 4% in women in the general population [3,4]. If untreated, OSA can cause morning headaches, fatigue, excessive daytime drowsiness, and cognitive impairment [5]. Moreover, OSA is an independent risk factor for cardiovascular, cerebrovascular, and metabolic disorders [6,7,8,9].

Anatomical abnormalities such as tonsils, adenoids, and polyps that constrict the upper airway warrant surgical therapy for OSA [10]. When conservative treatments including positive airway pressure devices, oral appliances, and positional therapy are unsuccessful, a surgical procedure might be considered [11]. The upper airway can be divided into several regions, including the nasal cavity, nasopharynx, oropharynx, and hypopharynx. Among these structures, the oropharynx is the region most frequently obstructed anatomically in OSA patients [12]. Thus, it is the most frequently targeted site for various surgical procedures targeting the palate and the pharyngeal wall, especially the lateral pharyngeal wall [12,13].

Classically, over many past decades, many physicians have performed uvulo-palato-pharyngoplasty (UPPP) for the treatment of OSA, which includes surgically excising the palatine tonsils as well as fully resecting the uvula and partially resecting the soft palatal tissues and lateral pharyngeal wall mucosa and muscular tissues [13]. Although it is stated in many pieces of literature that UPPP is shown to improve subjective symptom improvement related to OSA, the surgical results of UPPP have shown unpromising results as less than half of patients who have undergone UPPP showed no improvement following UPPP in polysomnography studies [13]. Moreover, many surgeons have reported that patients who have received UPPP may suffer from irreversible yet unpredictable complications such as velopharyngeal insufficiencies, nasopharyngeal stenosis, and sensation abnormalities of the palatal area, which can be very bothersome and generates huge frustration both to patients and surgeons [14]. Therefore, since the 2000s, many variations of UPPP have been proposed by many surgeons, respecting the function and preserving the oropharyngeal and soft palate structures, especially the uvula, but at the same time expanding and preventing collapsing of the palatal structures during sleep [15].

One of the efficient surgical methods to overcome this issue is the “limited palatal muscle resection (limited PMR)” described in many previous publications [14,15,16]. The surgical procedure known as the “limited palatal muscle resection (PMR)” entails the removal of a small section of the palatal muscle located at the soft palate [15]. This procedure has been shown to reduce respiratory disturbances by decreasing the volume and increasing muscle tension in the soft palate [15]. The overall efficacy of limited PMR as a surgical method for alleviating breathing disruptions during sleep is uncertain, despite positive results from individual studies [16,17,18,19]. Nonetheless, no meta-analysis has yet assessed the relationship between limited PMR and the occurrence of respiratory events during sleep. Therefore, the authors aimed to conduct a meta-analysis of existing literature to examine the efficacy of limited PMR for treating OSA.

## 2. Materials and Methods

### 2.1. Ethical Declaration

This study was approved by the institutional review board (IRB) of Inha University Hospital (investigation No.: 2023-06-002). Since this investigation was conducted by analyzing previously published literature that does not include any identifiable information about patients or individuals, the IRB waived informed consent.

### 2.2. Search Strategy

A comprehensive literature search was conducted using multiple databases, PubMed (Medline), EMBASE, Cochrane Library, Web of Science, KoreaMed, KSI, and KISS, to investigate the effectiveness of limited PMR in the treatment of OSA. Keywords used for the search were OSA-related index words (“obstructive sleep apnea*” OR “sleep apnea-hypopnea syndrome” OR “upper airway resistance sleep apnea syndrome” OR “obstructive sleep apnea syndrome*” OR “obstructive sleep hypopnea*” OR “obstructive sleep-disordered breathing”), palate muscle resection-related index words (“palatal muscle*” AND (“resection*” OR “resectab*” OR “dissection*” OR “excision*” OR “ablation*” OR “remov*”)) (Supplementary Tables S1–S3).

### 2.3. Eligibility Criteria and Study Selection

The present meta-analysis included original articles that assessed respiratory disturbances before and after limited PMR in patients with sleep-disordered breathing. The included studies had varying designs, such as retrospective studies, prospective (non-randomized) studies, and randomized controlled trials. However, studies were excluded if respiratory disturbances data such as the apnea-hypopnea index (AHI), the respiratory disturbance index (RDI), and/or lowest pulse oximetry values (minimum SpO_2_) were not clearly provided before or after a surgical procedure or if they lacked the necessary data essential for conducting a meta-analysis.

Following an independent screening of titles and abstracts of all possible articles by two authors (Lee and Choi), clinical studies that did not meet the criteria or were deemed irrelevant were excluded from the final analysis. There were no language restrictions imposed on papers included in this study. Selected articles underwent a meticulous and comprehensive review process.

### 2.4. Data Extraction

Data from chosen articles were collected using standardized forms. Extracted data consisted of the total number of subjects, AHI (events/hour), outcome measures, level of evidence (study design), language, and various demographic data such as age (years), sex (male: female), and body mass index (BMI; kg/m^2^).

Quality assessment was performed for non-randomized controlled studies based on the Newcastle–Ottawa Scale (NOS) and then converted to the Agency for Healthcare Research and Quality (AHRQ) standards (good, fair, and poor) [20,21]. The NOS is an evaluation tool comprising multiple criteria grouped into three main categories: selection of study groups, comparability of groups, and ascertainment of the outcome of interest. Each criterion is assigned a specific star rating, reflecting its importance and impact on the overall quality assessment. The scale utilizes a system of up to 9 stars to indicate the quality of the research. For good quality, the criteria are having 3 or 4 stars in the selection domain, 1 or 2 stars in the comparability domain, and 2 or 3 stars in the outcome/exposure domain. Fair quality is indicated by 2 stars in the selection domain, 1 or 2 stars in the comparability domain, and 2 or 3 stars in the outcome/exposure domain. Poor quality is defined as having 0 or 1 star in the selection domain, 0 stars in the comparability domain, or 0 or 1 star in the outcome/exposure domain. If there were any differences or inconsistencies in opinions, the authors addressed them by engaging in discussions and reaching a consensus.

### 2.5. Statistical Analysis

We conducted comparisons for respiratory disturbances during sleep before and after limited PMR for OSA. Mean and standard deviation values of the AHI or RDI as well as the lowest SaO_2_ before and after intervention were collected from relevant articles.

Heterogeneity was assessed using both Cochran’s Q statistic test and the I-square test. The I-square test measures the degree of heterogeneity among studies beyond what could be expected by chance. Its values ranged from 0 (representing no heterogeneity) to 100 (representing maximum heterogeneity). Outcomes were presented with two-tailed *p*-values and 95% confidence intervals (CIs). In the case of significant heterogeneity among results (I^2^ > 50), the random effect model based on the DerSimonian–Laird method was applied. The underlying assumption of this model was that actual treatment effects in each clinical research could vary and follow a normal distribution. In case the heterogeneity was not significant (I^2^ < 50), we planned to apply a fixed-effect model. However, the fixed-effect model was not applied due to significant heterogeneity observed in all results. To identify publication bias, we employed both Egger’s test and a funnel plot. All analyses were conducted using Comprehensive Meta-analysis Version 2.0 (Biostat, Inc., Englewood, NJ, USA).

## 3. Results

**Figure 1:**
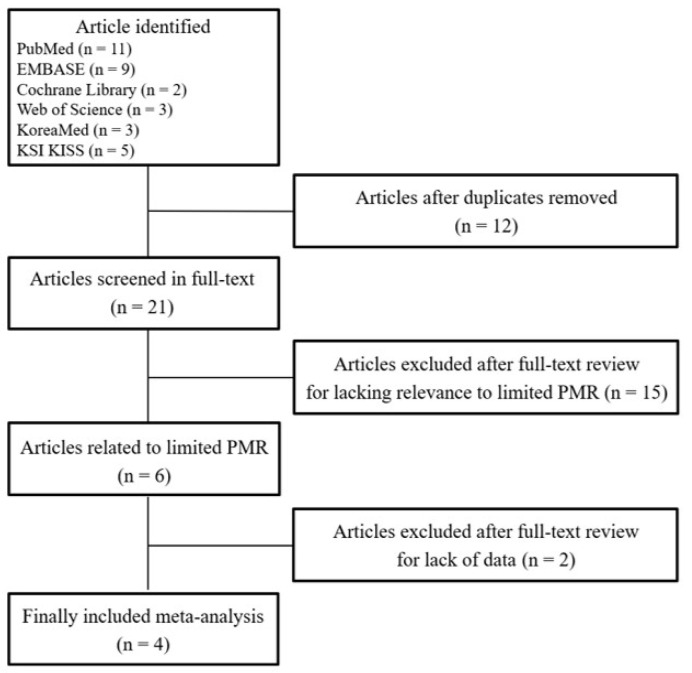
Preferred Reporting Items for Systematic Reviews and Meta-Analyses (PRISMA) flowchart. A PRISMA-compliant flow sheet has been developed to depict the present study. PMR, palatal muscle resection.

The PRISMA flowchart elaborating on the literature selection process is illustrated in Figure 1. Six articles examining respiratory disturbance-related parameters before and after limited PMR were retrieved for further review after screening their relevance. Two articles were excluded after a full-text review due to the lack of data. A total of four studies were finally included in this meta-analysis. Of these four studies, four showed data for the AHI or RDI and three showed data for minimum SpO_2_. Characteristics of these articles about the effectiveness of limited PMR in the treatment of OSA are outlined in Table 1. Effects of limited PMR on respiratory disturbances, including the AHI or RDI and minimum SpO_2_, are summarized in Table 2. The quality of these studies was assessed using the NOS and then converted to AHRQ standards. All studies were judged to be good for the risk of bias.

### 3.1. AHI/RDI before and after Limited PMR

**Figure 2:**
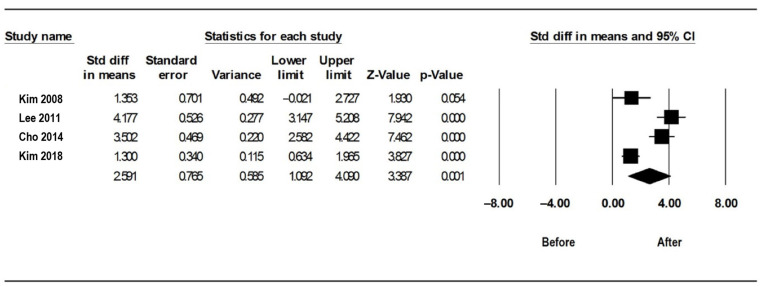
Forest plot for the effect of limited palatal muscle resection (PMR) on the apnea-hypopnea index (AHI)/respiratory disturbance index (RDI) [14,15,18,19]. A significant improvement in the AHI or RDI following limited PMR is depicted. Each black square represents the outcome of a particular study, while the horizontal lines represent the 95% confidence intervals for each study’s results, with each line’s end representing the confidence interval’s (CI) boundaries. The black diamond represents the overall result when the results of all studies are combined and averaged. The longitudinal points of the diamond depict the 95% CI limits. CI, confidence interval; Std diff, standardized difference.

**Figure 3:**
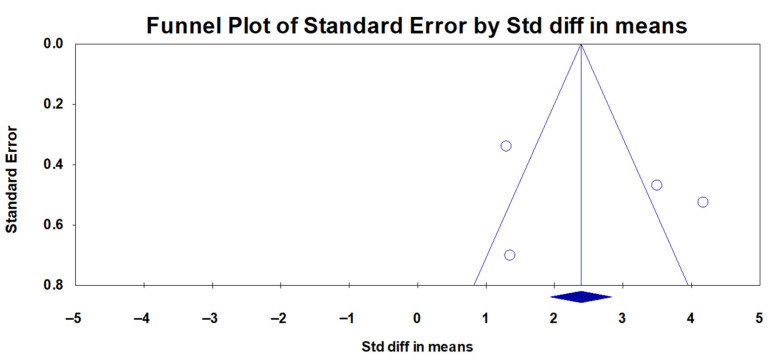
Funnel plot for the effect of limited palatal muscle resection on the apnea-hypopnea index (AHI)/respiratory disturbance index (RDI). A funnel plot analyzing the publication bias in terms of the AHI or RDI in the searched literature is depicted and carefully suggests lower evidence of a significant publication bias as an asymmetrical distribution of the plotted dots is not observed in the depicted illustration. The circles represent each individual study, while the blue diamond represents the genuine effect estimate that has been adjusted for publication bias. Std diff, standardized difference.

A total of four studies were included in the analysis of the AHI or RDI. In all four studies, the AHI or RDI decreased after surgery compared to that before surgery, although one study did not show a significant decrease. The I^2^ value was 89.9, indicating significant heterogeneity. Thus, a random effect model was used. The 95% CI of the mean differences ranged between 1.092 and 4.090, indicating a significance in the reduction in the AHI/RDI (Figure 2). The p-value from the Egger test was 0.600, and the funnel plot showed a symmetrical distribution, indicating no publication bias (Figure 3).

### 3.2. Minimum SpO_2_ before and after Limited PMR

**Figure 4:**
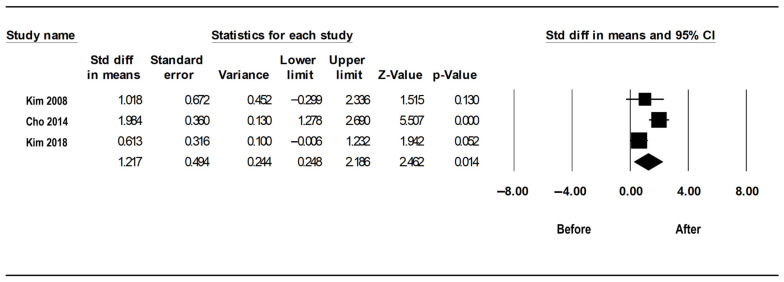
Forest plot for the effect of limited palatal muscle resection (PMR) on the minimum SpO_2_ (minimum SpO_2_) [14,18,19]. Following limited PMR, a significant increase in the minimum SpO_2_ is noticeable. Each black square represents the outcome of a particular study, while the horizontal lines represent the 95% confidence intervals for each study’s results, with each line’s end representing the confidence interval’s (CI) boundaries. The black diamond represents the overall result when the results of all studies are combined and averaged. The longitudinal points of the diamond depict the 95% CI limits. Std diff, standardized difference; CI, confidence interval.

**Figure 5:**
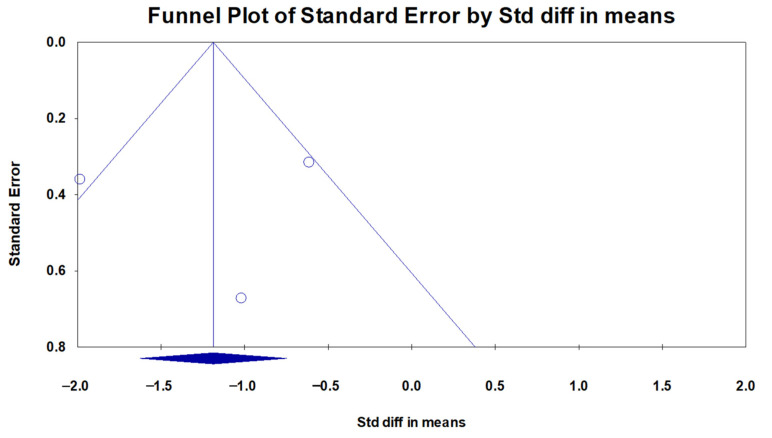
Funnel plot for the effect of limited palatal muscle resection on minimum SpO_2_ (minimum SpO_2_). A funnel plot analyzing the publication bias in the minimum SpO_2_ in the searched literature is depicted. Although the number of dots is very low, the arrangements of these dots show symmetrical distribution, thereby carefully suggesting a minimal possibility of publication bias. The circles represent each individual study, while the blue diamond represents the genuine effect estimate that has been adjusted for publication bias. Std diff, standardized difference.

A total of three studies were included in the analysis of the lowest SpO_2_ level. In all three studies, the minimum SpO_2_ increased after surgery compared to that before surgery, although only one study showed a statistically significant increase. The I^2^ value was 75.8, indicating significant heterogeneity. Thus, a random effect model was used. The standardized mean difference (95% CI) was 1.217 (0.248–2.186), indicating a significant increase in minimum SpO_2_ (Figure 4). The p-value from the Egger test was 0.936, and the funnel plot showed a symmetrical distribution, indicating no publication bias (Figure 5).

## 4. Discussion

Limited PMR, a surgical method of performing a tight anastomosis after partially resecting the palatine muscle involved in lifting the soft palate, was first reported by Kim et al. [14] in 2008 to overcome the fatal flaws but also to show better surgical results of the classical surgical method, UPPP [14]. Some of the fatal demerits of classical UPPP procedures are sensational abnormalities in the oropharyngeal region and velopharyngeal insufficiencies following surgery. These complications can be often irreversible once they take place following surgery, and the patients may be very frustrated and suffer from these complications permanently [18].

The results of this new surgical method, limited PMR, showed promising results compared with UPPP [15,16]. Briefly, this new surgical method is designed to improve respiratory disturbances during sleep by increasing the tension of muscles and widening the space of the upper airway, as well as to focus on preserving the important structure that is responsible for humidification and swallowing, which are represented to be the uvula and soft palatal mucosal tissues [18]. The essence of this limited PMR is to preserve the important anatomical structure, which is the uvula and soft palate mucosa. For more detailed descriptions of the surgical steps and procedures, detailed illustrations are depicted in Cho et al.’s publication from 2014 [18].

Our meta-analysis was designed to estimate how limited PMR could affect respiratory disturbances, including the AHI / RDI and min SaO_2_, through a systemic literature search and meta-analysis. Results of the present study revealed a decrease in the AHI and an increase of minimum SpO_2_ in OSA patients who underwent limited PMR. This was assessed in a total number of 21 patients who were evaluated for excessive daytime sleepiness using the Epworth Sleepiness Scale (ESS) and postoperative pain using the Visual Analogue Scale (VAS). Polysomnography was performed before and after surgery for five patients. ESS decreased significantly from 10.7 to 6.7 after surgery. In addition, the RDI (events/hour) decreased from 12.3 to 4.3 and the lowest SaO_2_ (%) increased from 89.8 to 93.6 after surgery compared to the results of polysomnography before surgery.

In 2011, Lee et al. [15] conducted uvulopalatal flap (UPF; *n* = 20) and limited PMR (*n* = 23) for patients with OSA and compared changes in subjective symptoms with results of polysomnography according to the surgical method. In a subjective symptom assessment using VAS before and after surgery, snoring, sleep apnea, tiredness, and daytime sleepiness were significantly reduced in both the UPF group and the limited PMR group. In the comparative analysis of polysomnography before and after surgery, the AHI (events/hour) improved from 25.9 preoperatively to 10.6 postoperatively in the UPF group and from 42.0 preoperatively to 5.6 postoperatively in the PMR group. However, although the min SaO_2_ and the mean SpO_2_ during sleep were alleviated after surgery, preoperative and postoperative results of oxygen saturation showed no significant difference in either group. These results suggest that limited PMR is an effective surgical procedure for managing OSA despite being a less invasive method than UPF.

In 2012, Koo et al. [16] evaluated the clinical safety of limited PMR in patients with OSA (*n* = 14) based on postoperative pain, voice analysis, the Eustachian tube function test, and the measurement of forced expiration power. As a result, postoperative pain was minimal. There was no noticeable alteration in vowel sounds including /u/. There were no signs of postoperative nasalization in the voice, Eustachian tube malfunction, or alterations in expiration power either. These outcomes indicate that limited PMR might be considered a clinically safe surgical procedure for patients with OSA.

To investigate the effectiveness of limited PMR with or without uvulectomy, Kim et al. [17] compared postoperative outcomes between two groups: limited PMR alone (*n* = 17) and limited PMR with partial uvulectomy (*n* = 16) in patients with OSA. Postoperative subjective symptoms using VAS such as snoring, sleep apnea, and daytime sleepiness were significantly improved in both groups. There were no significant differences in subjective symptoms between the two groups after surgery. Although dry throat and foreign body sensation were significantly increased at 1 and 4 weeks postoperatively in the limited PMR with partial uvulectomy group compared to those in the limited PMR group in the evaluation of postoperative complications, these differences were resolved within three months.

In 2014, Cho et al. [18] evaluated the effect of limited PMR with tonsillectomy on various sleep-disordered breathing parameters including subjective symptoms and polysomnographic data in patients with OSA (*n* = 23). Magnetic resonance images (MRIs) were compared before and after surgery for 10 patients [18]. Six months after surgery, there were significant improvements in subjective symptoms such as snoring, morning headache, tiredness, and daytime sleepiness, as well as polysomnographic data including AHI and cumulative proportion of total sleep time spent with SaO_2_ below 90% (CT90). In addition, there was a significant decrease in the soft palate length from 28.3 ± 2.3 to 24.5 ± 1.9 mm along with a significant increase in the distance between the tip of the uvula and the posterior pharyngeal wall from 5.1 ± 1.1 to 10.2 ± 1.5 mm, resulting in an upward and anterior movement of the uvula and soft palate in the postoperative MRI after surgery. Moreover, no patients exhibited symptoms of velopharyngeal insufficiency, voice alterations, or dryness in the pharynx at 6 months follow-up. These results show that limited PMR is a safe and effective technique for treating OSA in patients with oropharyngeal obstruction, resulting in significant improvements in both subjective and objective OSA parameters while maintaining pharyngeal function.

To identify factors that impacted the success rate of limited PMR, Kim et al. [19] utilized drug-induced sleep endoscopy (DISE) to evaluate its potential as a predictor of therapeutic response in OSA patients. A total of 21 consecutive OSA patients who received limited PMR underwent evaluations based on clinical data, polysomnographic parameters, cephalometric variables, and DISE findings, along with preoperative and postoperative AHI to determine success or failure of limited PMR. After surgery, there were significant alleviations in postoperative AHI and minimum SpO_2_. The overall success rate of limited PMR was 66.6%. Although there was no significant difference in BMI, Friedman stage, preoperative AHI, lowest O_2_, or cephalometric parameters between success and failure groups, the success of limited PMR was significantly associated with the site, degree, and configuration of collapse observed during DISE. A complete collapse in the velopharynx with an anterolateral or concentric pattern had a significantly higher success rate than a partial collapse with a lateral pattern. These outcomes suggest that DISE is the only predictive tool for determining the success of limited PMR in patients with OSA. Those with retropalatal narrowing, complete obstruction in the velopharynx, and an anteroposterior or concentric pattern might be considered appropriate candidates for the procedure. In the management of OSA patients, conducting a comprehensive evaluation of factors associated with surgical success for each surgical procedure and subsequently selecting the most suitable surgical procedure are considered highly important [22].

Interestingly, the latest publication in the systemic literature search discussing the outcomes of limited PMR was from 2018, and none have been published since then. We believe that there are some interesting socioeconomic backgrounds in regard to the matter, which is a very interesting aspect to discuss. From 2018, the Korean National Health Insurance System (KNHIS) has issued that the usage of a positive airway pressure device (PAP) shall be covered for all Korean citizens who show a necessity of treatment of OSA as proven in the full-night level I in-laboratory polysomnography studies. (i.e., moderate to severe degree of OSA or mild OSA with cardiovascular comorbid diseases) [23]. In contrast, the insurance coverage for surgical treatment of OSA did not change. In addition to that, there has been an increased number of evidence that PAP devices show more advanced efficacy with fewer complications compared to the surgical treatment of OSA, especially in that era, up until recently [23,24,25]. Thereby, we believe that these factors might have influenced many physicians on their practice trends for the treatment of OSA around 2018 until now, which eventually might have led to a decrease in the publication of, or clinical studies on, surgical treatments for OSA, including limited PMR.

Although this systemic analysis concluded the efficacy of limited PMR in OSA patients, there are several limitations to be acknowledged. First, it should be acclaimed that parameters representing the degree of OSA may not be derived from level I polysomnography sleep studies in some of the included literature. Additionally, some studies have presented the AHI to represent the severity of OSA, while others used the RDI, which might be a major limitation in our results since the AHI and RDI both are used to represent OSA severity, but there are some differences between those two parameters. Second, the limitation of this meta-analysis study is the relatively small number of published studies available reporting or discussing limited PMR. Moreover, due to the small number of included studies for the analysis, we were not able to register our systemic analysis in the Prospective Register of Systematic Reviews (PROSPERO) [26]. We suggest that with more publications, a future systemic analysis can be conducted following registration in the PROSPERO in the future. Furthermore, although we have carefully suggested that there is a minimal possibility of publication bias in our results, this should be addressed due to the very limited number of studies included for analysis. In a systemic review or meta-analysis, publication bias is the failure to publish a study’s results based on the direction or intensity of the study’s findings [27]. To determine the presence of publication bias, depicting an asymmetrical distribution of collected data in a funnel plot is a helpful way to assess this regard [28]. A funnel plot is an illustration of the relationship between an experiment’s effect magnitude and its precision, which is depicted as a scatter plot [28]. In the funnel diagram, the estimated treatment effects from individual studies (horizontal axis) are plotted against the sample size (vertical axis). Publication bias is indicated by asymmetries in the funnel diagram, as determined by regression analysis [27]. In the absence of bias and heterogeneity, funnel diagrams ought to be funnel-shaped and symmetrically centered around the analyses summary effect estimate [27,28]. As our funnel diagram depicted in Figure 3 and Figure 4 demonstrates a relatively symmetrical distribution of the four included pieces of literature, we can cautiously conclude that publication bias is not that prevalent. However, since only four studies were included in our analysis, this bias should be validated in future research when a larger sample size is available. Third, the term “limited PMR” may not be universally adopted, as some surgeons in different areas of the world or that speak different languages may describe the same or similar surgical procedure in another terminology or may include this procedure among the term “palatoplasty”, which might have resulted in a limited number of literature inclusion. Also, the fact that the previous research included for analysis was conducted mainly in a specific region of the world (South Korea) could very much affect the outcomes of the results of the systemic analysis. Fourth, despite limited PMR showing its efficacy in improving the AHI and minimum SpO_2_ levels, other sleep-related questionnaires or patient-reported outcome measurements regarding the quality of life were inconsistent in the published literature for a meta-analysis in our studies. Lastly, we were not able to perform a subgroup analysis and clarify which individuals might most benefit from limited PMR and those who may not. In future studies with added publications on limited PMR, it would be an interesting area of investigation to elucidate the ideal candidates and thus establish a precise indication for limited PMR in upcoming studies.

## 5. Conclusions

This study provides significant evidence that limited PMR is effective in reducing AHI and improving SpO_2_ levels in patients with OSA. However, additional research on limited PMR is necessary, especially with a focus on selecting individuals who might benefit the most from this surgical method and those who would most likely benefit less. Nevertheless, it is crucial to regard it as a noteworthy surgical alternative for OSA patients who fulfill the criteria for surgical intervention. This meta-analysis enhances our understanding of the effects of restricted PMR on OSA treatment.

## Figures and Tables

**Table 1:** Characteristics of studies on the effectiveness of limited PMR in the treatment of OSA.

Author	Published Year	Total Number of Subjects	Age (Years)	Sex (M:F)	BMI (kg/m²)	AHI (RDI) (Events/h)	Outcome Measures	Level of Evidence (Study Design)	Language
Studies included in the final analysis									
Kim et al. [14]	2008	21	19~70	19:2	24.5	RDI ≤ 20	ESS, AI, RDI, mean SaO_2_, lowest SaO_2_, postoperative pain (VAS)	Level IV (retrospective)	Korean
Lee et al. [15]	2011	23	36.8 ± 8.7	20:3	24.5 ± 3.1	42.0 ± 9.7	ESS, snoring (VAS), sleep apnea (VAS), morning headache (VAS), tiredness (VAS), daytime sleepiness (VAS), AHI, mean SaO_2_, lowest SaO_2_	Level IV (retrospective)	Korean
Cho et al. [18]	2014	23	36.8 ± 8.7	20:3	24.5 ± 5.6	32.0 ± 10.2	ESS, snoring (VAS), morning headache (VAS), tiredness (VAS), daytime sleepiness (VAS), AHI, mean SaO_2_, lowest SaO_2_, CT90, soft palate length, retropalatal space	Level II (prospective)	English
Kim et al. [19]	2018	21	45.2 ± 11.0	20:1	25.7 ± 2.8	33.6 ± 19.0	AHI, lowest SaO_2_, CT90, cephalometric data	Level II (prospective)	English
Studies discussing the efficacy of limited PMR but not included in the final analysis due to the lack of data									
Koo et al. [16]	2012	14	37.0 ± 7.12	14	25.4 ± 0.7	13.3 ± 3.6	ESS, postoperative pain (VAS), Eustachian tube function test, voice analysis, measurement of forced expiration power	Level IV (retrospective)	Korean
Kim et al. [17]	2014	17 (PMR alone)	41.9 ± 11.7	11:6	24.1 ± 3.8	17.4 ± 17.5	Snoring (VAS), sleep apnea (VAS), daytime sleepiness (VAS), postoperative complications (VAS)	Level IV (retrospective)	Korean
		16 (PMR with uvulectomy)							

M, male; F, female; BMI, body mass index; AHI, apnea-hypopnea index; RDI, respiratory disturbance index; PMR, palatal muscle resection; ESS, Epworth sleepiness scale; AI, apnea index; VAS, visual analogue scale; CT90, cumulative time spent with SaO_2_ below 90%.

**Table 2:** Effect of limited PMR on respiratory disturbances including AHI/RDI and lowest oxygen saturation on a pulse oximeter (minimum SpO_2_).

Author	Published Year	Final Number of Patients	Preoperative AHI (RDI) (Events/h)	Postoperative AHI (RDI) (Events/h)	Preoperative Minimum SpO_2_ (%)	Postoperative Minimum SpO_2_ (%)
Kim et al. [14]	2008	5	12.3 ± 7.5	4.3 ± 3.7	89.8 ± 5.2	93.6 ± 0.9
Lee et al. [15]	2011	23	42.0 ± 9.7	5.6 ± 7.6	88.3	NA
Koo et al. [16]	2012	14	13.3 ± 3.6	NA	85.6 ± 2.3	NA
Kim et al. [17]	2014	17 (PMR alone)	17.4 ± 17.5	NA	NA	NA
		16 (PMR with uvulectomy)	31.6 ± 34.2	NA	NA	NA
Cho et al. [18]	2014	23	32.0 ± 10.2	5.6 ± 3.1	83.2 ± 3.1	88.3 ± 1.9
Kim et al. [19]	2018	21	33.6 ± 19.0	12.8 ± 12.3	78.4 ± 8.6	83.1 ± 6.6

PMR, palatal muscle resection; AHI, apnea-hypopnea index; RDI, respiratory disturbance index; NA, not applicable; Minimum SpO_2_, minimum SpO_2_.

## Data Availability

The datasets used and/or analyzed during the current study are available from the corresponding author on request.

## Supplementary Materials

The following supporting information can be downloaded at https://www.mdpi.com/article/10.3390/medicina59081432/s1. Table S1: Search strategies (search queries) in PubMed. Table S2: Search strategies (search queries) in EMBASE. Table S3: Search strategies (search queries) for Cochrane Library.
