# Supplementary material for: Limited Palatal Muscle Resection for the Treatment of Obstructive Sleep Apnea: A Systematic Review and Meta-Analysis
Source: Medicina (Kaunas). 2023 Aug 8;59(8):1432. doi: 10.3390/medicina59081432 (PMC10456771; doi:10.3390/medicina59081432)
Supplement: Supplementary file 1 [file medicina-59-01432-s001.zip › medicina-2489438-supplementary.pdf]

Supplementary Materials

Table S1: Search strategies (search queries) in PubMed.

No	Search Queries	Searched Literatures (*n*)
1	"sleep apnea, obstructive"[MeSH Terms]	26,639
2	"obstructive sleep apnea*"[Title/Abstract] OR "sleep apnea hypopnea syndrome"[Title/Abstract] OR "upper airway resistance sleep apnea syndrome"[Title/Abstract]	30,154
3	**1 OR 2**	**38,149**
4	"sleep apnea syndromes"[MeSH Terms]	42,688
5	"sleep apnea syndrome*"[Title/Abstract] OR "sleep hypopnea*"[Title/Abstract] OR "sleep disordered breathing"[Title/Abstract]	15,836
6	**4 OR 5**	**46,471**
7	"obstructi*"[Title/Abstract]	316,141
8	**6 AND 7**	**30,329**
9	**3 OR 8**	**40,670**
10	"palatal muscles"[MeSH Terms]	640
11	"palatal muscle*"[Title/Abstract]	101
12	**10 OR 11**	**691**
13	"resection*"[Title/Abstract] OR "resectab*"[Title/Abstract] OR "dissection*"[Title/Abstract] OR "excision*"[Title/Abstract] OR "ablation*"[Title/Abstract] OR "remov*"[Title/Abstract]	1,403,757
14	**12 AND 13**	**73**
15	**9 AND 14**	**11**
16	**15** NOT ("animals"[MeSH Terms:noexp] NOT ("animals"[MeSH Terms:noexp] AND "humans"[MeSH Terms]))	**11**

Table S2: Search strategies (search queries) in EMBASE.

No	Search Queries	Searched Literatures (*n*)
1	sleep disordered breathing'/exp	98,560
2	obstructive sleep apnea*':ti,ab OR 'sleep apnea hypopnea syndrome':ti,ab OR 'upper airway resistance sleep apnea syndrome':ti,ab	48,370
3	**1 OR 2**	**101,069**
4	sleep apnea syndrome*':ti,ab OR 'sleep hypopnea*':ti,ab OR 'sleep-disordered breathing':ti,ab	24,113
5	obstructi*':ti,ab	454,990
6	**4 AND 5**	**14,843**
7	**3 OR 6**	**101,234**
8	palate muscle'/exp	102
9	palatal muscle*':ti,ab	126
10	**8 OR 9**	**207**
11	resection*':ti,ab OR 'resectab*':ti,ab OR 'dissection*':ti,ab OR 'excision*':ti,ab OR 'ablation*':ti,ab OR 'remov*':ti,ab	1,886,338
12	**10 AND 11**	**33**
13	**7 AND 12**	**9**
14	**13** NOT ('animal'/de NOT ('animal'/de AND 'human'/exp)) (Limitation)	**9**

Table S3: Search strategies (search queries) for Cochrane Library.

No	Search Queries	Searched Literatures (*n*)
1	MeSH descriptor: [Sleep Apnea, Obstructive] explode all trees	2,616
2	(obstructive sleep apnea*):ti,ab,kw OR (sleep apnea hypopnea syndrome):ti,ab,kw OR (upper airway resistance sleep apnea syndrome):ti,ab,kw	6,207
3	**1 OR 2**	**6,258**
4	MeSH descriptor: [Sleep Apnea Syndromes] explode all trees	3,419
5	(sleep apnea syndrome*):ti,ab,kw OR (sleep hypopnea*):ti,ab,kw OR (sleep-disordered breathing):ti,ab,kw	6,537
6	**4 OR 5**	**7,110**
7	(obstructi*):ti,ab,kw	43,217
8	**6 AND 7**	**5,418**
9	**3 OR 8**	**6,575**
10	MeSH descriptor: [Palatal Muscles] explode all trees	14
11	(palatal muscle*):ti,ab,kw	62
12	**10 OR 11**	**62**
13	(resection*):ti,ab,kw OR (resectab*):ti,ab,kw OR (dissection*):ti,ab,kw OR (excision*):ti,ab,kw OR (ablation*):ti,ab,kw OR (remov*):ti,ab,kw	89,575
14	**12 AND 13**	**16**
15	**9 AND 14**	**2**
16	**15** NOT ("animals"[MeSH Terms:noexp] NOT ("animals"[MeSH Terms:noexp] AND "humans"[MeSH Terms:noexp]))	**2**
